# Acceleration of differentiated service delivery for HIV treatment in sub‐Saharan Africa during COVID‐19

**DOI:** 10.1002/jia2.25704

**Published:** 2021-06-09

**Authors:** Anna Grimsrud, Lynne Wilkinson

**Affiliations:** ^1^ HIV Programmes and Advocacy International AIDS Society Cape Town South Africa; ^2^ Centre for Infectious Disease Epidemiology and Research University of Cape Town Cape Town South Africa

**Keywords:** HIV, ART, differentiated service delivery, COVID‐19, multi‐month dispensing, community‐based services

## Abstract

**Introduction:**

In response to COVID‐19, national ministries of health adapted HIV service delivery guidelines to ensure uninterrupted access to antiretroviral therapy (ART) and limit the frequency of contact with health facilities. In this commentary, we summarize four ways in which differentiated service delivery (DSD) for HIV treatment has been accelerated during COVID‐19 in policy and implementation in sub‐Saharan Africa (SSA) – (i) expanding eligibility for DSD for HIV treatment, (ii) extending multi‐month dispensing (MMD) and reducing the frequency of clinical consultations, (iii) emphasizing community‐based models and (iv) integrating/aligning with TB preventative therapy (TPT), non‐communicable disease (NCD) treatments and family planning commodities.

**Discussion:**

Across SSA in 2020, countries both adapted and emphasized policies supporting DSD for HIV treatment in response to COVID‐19. Access to DSD for HIV treatment was expanded by reducing the time required on ART before eligibility and being more inclusive of specific populations including children and adolescents, pregnant and breastfeeding women and those on second‐ and third‐line regimens. Access to extended ART refills, or MMD, was accelerated across many countries. A renewed focus was given to out‐of‐facility community‐based models of ART distribution. In some settings, there was acknowledgement of the need to integrate or align other chronic medications with ART.

**Conclusions:**

Adaptations to DSD for HIV treatment in response to COVID‐19 have resulted in rapid policy change and in some cases, acceleration of implementation in SSA. As the COVID‐19 pandemic evolves, there is a critical need to assess the impact of these adaptations and, where beneficial, ensure that policies implemented in response to COVID‐19 become the new normal.

## 1 Introduction

On World AIDS Day 2020, UNAIDS announced that none of the global HIV targets – 90% of people living with HIV know their status, 90% of those are receiving antiretroviral therapy (ART) and 90% of those on ART are virually suppressed‐ would be reached by the end of the year [1]. Modelling work published in August 2020 emphasized the potentially catastrophic impact on AIDS‐related mortality and HIV transmission if there was a six‐month interruption in ART in Africa [2]. Programmatic data confirmed that while the global 2020 HIV targets would not be met, the total number of people on ART did not decline between April and September of 2020 [1]. The effect of COVID‐19 on HIV programmes has most severely impacted HIV prevention, testing and initiation of new patients, whereas HIV treatment programmes have been fairly resilient with 26 million people worldwide on treatment as of June 2020 [1].

Differentiated service delivery (DSD) for HIV treatment is an approach that puts the person at the centre and adapts services to meet their needs and expectations [3, 4]. In April 2020, we outlined how DSD for HIV treatment should be expedited and adapted in response to COVID‐19 – by reducing the frequency of visits and enabling ART refills outside of health facilities, expanding who was eligible for DSD for HIV treatment and supporting testing and rapid, community‐based initiation for those not on ART [5]. In addition, we emphasized the precedent for HIV programmes to adjust service delivery in emergency contexts. Nine months later, with data to support that in most countries the number of people on HIV treatment has been maintained despite COVID‐19, we can report on both DSD policy and implementation adaptations made by countries to facilitate uninterrupted treatment [1].

At the beginning of 2020 in sub‐Saharan Africa (SSA), access to DSD for HIV treatment was limited to people clinically stable on ART after six to twelve months of treatment. The duration of ART refills was mostly limited to one to three months. Specific populations, including children, adolescents and pregnant and breastfeeding women, as well as people living with HIV and other comorbidities were largely excluded from accessing DSD. Furthermore, TB preventive therapy (TPT) and family planning commodities were not aligned or integrated within DSD for HIV treatment models and key populations continued to have limited access [6, 7].

Between March and August 2020, many ministries of health in SSA, with support from PEPFAR and the Global Fund, issued national guidelines on how to adapt HIV programmes in response to COVID‐19 to both facilitate uninterrupted ART provision and reduce contact with health facilities to minimize the risk of exposure to SARS‐CoV‐2 infection for people living with HIV and healthcare workers [8, 9, 10, 11, 12, 13, 14, 15, 16, 17, 18, 19, 20, 21, 22, 23, 24, 25, 26, 27, 28, 29, 30, 31, 32, 33, 34, 35, 36]. As a result, the COVID‐19 pandemic response resulted in many previous DSD policy barriers being removed, at least temporarily.

We reviewed national interim guidance provided for HIV service delivery during COVID‐19 across SSA documenting policy adaptations. In addition, data shared through webinars, virtual conferences and from partners were assessed to highlight the implementation of these policy adaptations. In this commentary, we summarize four ways in which DSD for HIV treatment has been accelerated during COVID‐19 in policy and implementation – (i) expanding eligibility for DSD for HIV treatment, (ii) extending multi‐month dispensing (MMD) and reducing the frequency of clinical consultations, (iii) emphasizing community‐based models and (iv) integrating/aligning with TPT, non‐communicable disease (NCD) treatments and family planning commodities.

## 2 Discussion

### 2.1 Expanding eligibility for DSD for HIV treatment

The 2016 recommendations from the World Health Organization (WHO) defined clinical stability as being on ART for 12 months or more with evidence of treatment success, ideally through viral load monitoring [37]. Before COVID‐19, many national policies had already reduced the time on ART requirement from twelve to six months. While WHO supported DSD access for specific populations [38], many were either explicitly excluded or not specifically made eligible in country policies [39]. PEPFAR, in response to COVID‐19, intensified their push for expanding eligibility to all populations, including those with co‐morbidities, those who recently initiated treatment and to those with advanced HIV disease, specifically at least for MMD [40].

#### 2.1.1 Policy

Many countries expanded eligibility by reducing or removing criteria related to time on ART before accessing DSD for HIV treatment, or just MMD (Table 1). In the Cote D’Ivoire [8], Democratic Republic of Congo (DRC) [9], Eswatini [10], Ethiopia [12], Liberia [18], South Africa [25], Togo [30], Uganda [31] and Zambia [34] all patients were eligible for MMD from ART initiation. In the DRC [9] and Mozambique [22] eligibility criteria for DSD for HIV treatment models changed to only three months on ART. In South Africa, new guidelines in March 2020 changed the eligibility criteria for DSD for HIV treatment models from two suppressed viral loads and 12 months on ART to one suppressed viral load and on ART for six months and included children above the age of five years and breastfeeding women unable to access fully integrated maternal and child health and ART care [41]. South Africa’s HIV guidance during COVID‐19 emphasized this new eligibility criteria for urgent implementation [25]. In Malawi and Mozambique, the criteria for a suppressed viral load was removed in response to COVID‐19, likely to overcome the challenge of poor [21, 22]. In Ethiopia, those on second‐ and third‐line regimens, clinically unstable patients and all children and pregnant and breastfeeding women became eligible for 3MMD during COVID‐19 [12]. Similarly, guidance in Eswatini during COVID‐19 included pregnant and breastfeeding women and children above the age of two years as eligible for 3MMD [10].

**Table 1:** National policy guidance adaptations to HIV service delivery in response to COVID‐19

	1. Expanding eligibility	2. Extending ART refills and prescriptions	3. Emphasize additional options including community‐based and/or extended clinic hours	4. Emphasized integration of other medications
Cote d'Ivoire [8, 60]	3MMD for all populations from ART start regardless of viral load, or advanced HIV disease	No change	Yes – Home delivery for those over 60 years of age	Synchronize medications, particularly TPT
Democratic Republic of Congo [9, 61]	3MMD for all populations from ART start. Patients on ART for 3MMD with no contraindications can receive 6MMD	Change from 3MMD to 6MMD		
Eswatini [10, 62]	3‐6MMD for all populations from ART start. 3MMD for specific populations (children above 2, PBFW) and for 2^nd^‐line clients on DTG (other 2^nd^‐line clients still 1MMD).	Change from the maximum of 3MMD to 6MMD	Yes – Community Adherence Groups can pick‐up medication refills for community – inclusive of TB, TPT, NCDs, family planning.	MMD co‐administered with TPT, cotrimoxazole (CTX), fluconazole and contraceptive commodities
Ethiopia [12, 63]	3MMD from ART start including for PMTCT, paediatrics, clients on second and third‐line regimens and those with advanced HIV disease	No change	Yes – Strengthen HCW‐managed community DSD models and PLHIV group‐managed DSD models to deliver ARTs at community sites, emphasized family collected refills, home delivery by implementing partners where feasible	MMD co‐administered with TPT, CTX, fluconazole and contraceptive commodities
Kenya [16, 64]	Expanded 3MMD for all ages (excluding PMTCT, newly diagnosed and not virally suppressed)^a^	No change	Yes – promote flexible ART delivery models such as community adherence groups and with staggered pick‐up times at health facilities	ART refills are aligned with TPT, CTX refills
Liberia [18, 19]	3MMD for all populations from ART start	Change from 3MMD to 6MMD (initially high volume then all sites)	Yes – Nurse‐led community dispensing, selected assisted community options for KPs	All medication refills to be aligned with ART refills
Malawi [21, 65, 66]		Prioritized 6MMD implementation		Efforts to align ART refills with TPT and CTX. Contraceptive commodities that are easily aligned were before COVID‐19.
Mozambique [22, 67]	Yes – 3MMD for all over 2 (including PBFW), no VL required, no active condition or WHO stage III/IV, including second and third‐line ART after three months on ART.	*No change*	Yes – Prioritize community adherence groups and expand access to mobile brigades	3MMD for TPT, CTX and other NCDs – integrated and offered in the same consultation room except for some of the contraceptives that require special care, NCD medication only 3MMD if stock allows
Sierra Leone [23, 68]	1‐3MMD from ART start for all populations	Extended from one to three months for stable adults and children	Yes – including home delivery, refills within support groups and refills from drop‐in centres for key populations	
South Africa [24, 25, 41, 69]	2MMD from ART start for all populations	Clinical consultations changed from 6 monthly to annually supported by prescriptions being extended to twelve months	Yes – focus on supporting clients to enrol in repeat prescription collection strategies, prioritizing external pick‐up points including transfer of patients from facility‐based to out‐of‐facility models. Home delivery	NCD refill already implemented pre‐COVID‐19 but prioritized. All medication refills to be aligned with ART refills.
Tanzania [28, 70]		6MMD implementation prioritization for adults in Dar es Salaam	Yes – scale up ART refills via treatment supporters, outreach and community group refill models	If on TPT regarded as unstable not eligible for DSD. Changed to alignment of TPT and CTX with ART refills.
Togo [30, 71]	3MMD for all populations from ART start			
Uganda [31, 72]	3MMD all populations from ART start (except visiting patients, on 2nd or 3rd line, viraemic, sick, lactating infant less than six months old)	No change	Yes – establish more community drug distribution points and expand client‐led ART delivery models	Alignment of TPT (six month refills with phone monitoring), TB and NCDs
Zambia [34, 73]	6MMD for all populations from ART start except 3MMD for two to ten years; viraemic. PLHIV with co‐morbidities 3‐6MMD.			
Zimbabwe [35, 74]		Extended 6MMD, 6MMD previously permitted for migrant workers	Yes – Strengthen family and community ART refill models	Policy on ART and TPT refills existed and was reiterated in the HIV and COVID‐19 guidance

^a^ Kenya updated interim guidance from 24 August 2020 revised this policy adaption to only “Where staff shortages, closure or relocation of service – 3MMD for all PLHIV regardless age/viraemia.”

#### 2.1.2 Implementation

Data on adaptations to eligibility in Ethiopia are available for children, pregnant women and for those newly initiated on treatment [42]. For children, the proportion of 3‐5MMD increased from 12% in October 2019 to 80% by July 2020. In the prevention of mother to child transmission data, there was an increase from 71% to 89% of women receiving 3‐5MMD (compared to <3MMD) between May and August 2020. Among those initiated on ART in the Addis Ababa region, at the beginning of May 2020 less than a third (31.6%) of patients received 3MMD at initiation and by mid‐July 2020, this had increased to 93.4%.

### 2.2 Extending the duration of ART refills and clinical consultations

WHO recommends three to six monthly ART refills and clinical consultations for people living with HIV who are clinically stable [37]. However, uptake of these policy recommendations is variable [43]. Additional gaps exist between policy and practice. During COVID‐19, many global stakeholders – UNAIDS [44], WHO [45], the Global Fund [46], UNICEF [47] – emphasized the importance of extending the duration of ART refills to ensure uninterrupted supply as countries imposed lockdowns and movement restrictions. Furthermore, recent trial data from Lesotho [48], Malawi and Zambia [49], South Africa [50] and Zimbabwe [51] corroborate non‐inferiority of 6MMD compared with shorter ART refills.

#### 2.2.1 Policy

In response to COVID‐19, many countries either extended the duration of ART refills or emphasized the maximum duration that had previously been specified but not broadly implemented (Table 1). In Sierra Leone [23], the maximum refill duration moved from one to three months. In Eswatini [10], Liberia [19] and South Sudan [27], the maximum duration moved to six months. In the DRC [9], Malawi [21] and Zimbabwe [35] where six‐month refills were already part of guidelines (in Zimbabwe limited to mobile populations), 6MMD was emphasized. Both Tanzania [28] and Zambia [34] focused on scaling up 6MMD with Tanzania’s focus on the Dar es Salaam region and Zambia prioritizing adolescents. In South Africa [24], the provision was made for the frequency of clinical consultations to be reduced to annually and for existing and new prescriptions written for ART to be extended from six to twelve months.

#### 2.2.2 Implementation

In 2020, across a number of countries the scaled implementation of extended ART refills has been remarkable. Eswatini started a 6MMD pilot in December 2019 achieving rapid expansion to 103 sites and almost 25,000 patients by March 2020 [personal communication]. Similarly, a Mozambiquan pilot became a national priority with 3MMD coverage extended to all 1,230 health facilities and growing from 39% in January 2020 to 66% by August 2020 [52]. In Tanzania, where 6MMD was limited to Dar es Salaam, less than 1% of adult patients were on 6MMD in February 2020 increasing to 29% by May 2020 [53]. Outside of Dar, 3MMD increased from 34% in April 2019 to 63% by April 2020. Malawi introduced 6MMD from April 2019 and by the end of September 2020, had nearly half of their treatment cohort on 6MMD, a total of 415,800 patients [54]. In Zambia, 6MMD was scaled to 56% of clients (n = 561,409) from July 2020, an increase from less than 50,000 in September 2019 [55]. Furthermore, 6MMD for children in Zambia increased to 25% up from 11% at the beginning of the year. PEPFAR global data, excluding South Africa, highlight this trend across its global programmes where 69% of PEPFAR‐supported clients received 3‐6MMD (16% on 6MMD) by the end of June 2020, up from 46% in December 2019 [55].

### 2.3 Extending community‐based ART delivery options

While it has been suggested at least 30% of ART delivery should be community‐based [1], both policies supporting this and investments in implementation had stalled. WHO estimates the percentage of countries with a policy promoting community‐based ART delivery has only increased from 21.1% to 22.5% between 2017 and 2020 [56]. COVID‐19 jumpstarted efforts, both from a policy and implementation perspective, emphasizing the benefits of expanding options for ART refills through extended clinic hours and out‐of‐facility models‐ both group, and individual dispensing models.

#### 2.3.1 Policy

Cote d’Ivoire [8], Eswatini [11], Ethiopia [12], Kenya [15], Lesotho [17], Mozambique [22], South Africa [25], South Sudan [27], Tanzania [28], Uganda [31] and Zimbabwe [35] all emphasized community‐based models for ART delivery within their HIV guidance during COVID‐19 (Table 1). Client‐led group models including Community Adherence Groups (CAGs) in Lesotho [17], Grupos de Apoio a Adesão Comunitária (GAACs) in Mozambique [22], community ART refill groups (CARGs) in Zimbabwe [35] and Community‐Client Led Adherence Delivery in Uganda [31] were leveraged to support the provision of uninterrupted ART supply with adaptations to support physical distancing and limited interactions with health facilities. Community drug distribution points were emphasized in Uganda [31] to increase the proportion of people collecting ART outside of health facilities. In Eswatini [11], adaptations to CAGs included CAG members being able to collect drug refills for their community (beyond just the people in their HIV CAG) including NCD, family planning and PrEP refills. Lay healthcare worker‐managed community groups were developed in Kenya [16], specifically for adolescents, young people and sex workers. Adaptations were made to support lay healthcare workers distributing ART refills to community groups, often with a virtual psychosocial support component.

In South Africa [25], where ART refills were mostly limited to two months, guidance emphasized the acceleration of external pick‐up points including ART refills from private pharmacies, community venues and lockers or “pele boxes.” Home‐delivery of ART was endorsed by policy in Cote Ivoire (prioritized for those over 60 years of age), Ethiopia, Sierra Leone and South Africa. Tanzania emphasized community‐based group models for adolescents.

#### 2.3.2 Implementation

In South Africa, the number of clients who received their ART through an external pick‐up point increased from 781,103 in 2019 to 1,313,384 by October 2020 [57]. In Tanzania, ICAP scaled‐up community ART refills from just 590 patients between July and September of 2019 to 20,089 in April and May 2020 [53]. Drop‐in centres for key populations in Sierra Leone [58] and Liberia [59] began providing ART refills.

Home delivery of ART refills was implemented both within countries with policy support and elsewhere through partnerships including with community‐based organizations and peer providers. In the Western Cape province of South Africa, 861,234 pre‐packed ART parcels were delivered via courier between April and November 2020 (personal communication).

### 2.4 Integrating TB preventive therapy, NCD treatment and family planning commodities

While the rationale for the integration of other preventive and therapeutic treatments within DSD for HIV treatment is clear, policy support and implementation data by the end of 2020 were limited.

#### 2.4.1 Policy

In HIV guidance in response to COVID‐19, a few countries (Cote D’Ivoire [8], Liberia [18] and South Africa [25]) emphasized the need to align refills for all medications among people living with HIV. Many countries emphasized the alignment of TPT and cotrimoxazole prophylaxis (CTX) (Table 1). In Eswatini [10], family planning integration with ART refills was highlighted. Provision was made for clients to access long‐acting injectables and for oral contraceptives refills to be aligned with ART refills. Similarly in Ethiopia [12], MMD of ART and oral contraceptives was emphasized.

NCD refill alignment was emphasized in the national policies of Mozambique [22] and Uganda [31]. Malawi [21] specifically excluded NCD refill alignment due to supply chain barriers and Eswatini stated concerns regarding decreasing the frequency of monitoring.

#### 2.4.2 Implementation

Data demonstrating integration implementation were not available.

### 2.5 Limitations

The data used for this synthesis were limited to what was publicly available and are likely incomplete. However, data gaps plausibly reflect inadequate reports and where DSD uptake was the slowest. While we do not have data on integration, it is unlikely that large data sources were missed. Furthermore, while there are data showing the resilience of the ART programme during COVID‐19 and data highlighting an increase in the number of people accessing DSD in 2020, there may not be a causal relationship.

## 3 Conclusions

By the end of 2020, access to DSD for HIV treatment had been expanded across countries in SSA to allow access to MMD from ART initiation before stability could be ascertained. People clinically stable on ART were entitled to access to DSD models from three to six months on ART. The duration of ART refills was widely extended with most countries providing 3MMD and many more people living with HIV accessing 6MMD when the supply chain allowed. Specific populations, including children, adolescents and pregnant and breastfeeding women as well as people living with HIV and other comorbidities also gained access to DSD for HIV treatment. Policies were updated to better align and/or integrate TPT and family planning commodities within DSD approaches. Access by key populations to DSD for HIV treatment was prioritized and expanded.

Accelerated DSD‐enabling policy and implementation have provided the necessary tools for ART programmes to survive the serious risks posed by the continuing COVID‐19 pandemic. This acceleration was necessary before COVID‐19 and continues to be critical to supporting long‐term retention of the growing number of people living with HIV on ART globally. However, remembering that DSD focuses on client preference and needs, COVID‐19 related DSD acceleration should not result in a new one‐size‐fits all approach of MMD and community individual refills. Choice to transition between HIV treatment service delivery models as needs change and evolve remains central.

It is now essential for countries to actively review interim policy changes and their implementation to determine which DSD adaptations are appropriate to continue beyond the COVID‐19 pandemic. It will also be critical to evaluate and generate evidence of the impact of these changes to inform global guidance and policy.

## Competing interests

The authors declare no conflict of interest.

## Authors’ contributions

AG and LW jointly developed the concept for the commentary. The review of national policies was led by LW and the review of implementation data by both LW and AG. AG wrote the first draft of the introduction and discussion and LW wrote the first draft of the conclusion. Both AG and LW approved the final version of the manuscript.

## Abbreviations

ART, antiretroviral therapy; CAGs, Community adherence groups; CTX, Cotrimoxazole prophylaxis; DRC, Democratic Republic of Congo; DSD, differentiated service delivery; GAACs, Grupos de Apoio a Adesão Comunitária; MMD, multi‐month dispensing; NCD, non‐communicable diseases; TPT, TB preventive therapy; WHO, World Health Organization.
